# Supplementary material for: Design dimensions of electrocutaneous warning stimulation patterns in workplace safety devices
Source: PLoS One. 2024 Dec 16;19(12):e0314554. doi: 10.1371/journal.pone.0314554 (PMC11649102; doi:10.1371/journal.pone.0314554)
Supplement: Attachment — Submitted filename: Review.pdf [file pone.0314554.s001.pdf]

This is an interesting study and the first impression about your article is stated below:

1-The manuscript is properly organized.

2- The abstract is well written and gives a reasonable summary for the article.

3- The introduction adequately covers the research issue

4- The researcher was successful in presenting the research methodology in detail, starting with the stages of preparing the subjects, the method of arranging the electrodes, and the method of applying the electrical signals in this study.

5-Before accepting the manuscript, in my opinion, there are few suggestions need to be considered with some notes require your explanation:

a- There is a need to include more previous studies on the topic
b- I suggest including a diagram that briefly explains the research methodology to facilitate the understanding for the reader.
c- I suggest including figure showing the arrangement of the electrodes.
d- You mention the optimal impedance in the section on preparation. Is it necessary for the impedance to be higher or lower, and if so, why? Could you please explain how you arrived at the optimal impedance?
