# Supplementary material for: Design dimensions of electrocutaneous warning stimulation patterns in workplace safety devices
Source: PLoS One. 2024 Dec 16;19(12):e0314554. doi: 10.1371/journal.pone.0314554 (PMC11649102; doi:10.1371/journal.pone.0314554)
Supplement: Attachment — Submitted filename: Rebuttal letter.pdf [file pone.0314554.s002.pdf]

**Rebuttal letter**

Dear Dr. Hadeel K. Aljobouri and Reviewers,

On behalf of my co-authors and myself, I extend our sincere thanks for your time and effort in reviewing our manuscript titled “Design dimensions of electrocutaneous warning stimulation patterns: pulse intervals, amplitude, and location”. Your detailed comments and constructive feedback have been very helpful in improving our work. We appreciate your thorough evaluations and professional guidance.

Please find our responses to your comments below.

Best regards,

E.-M. Dölker

Dr. Eva-Maria Dölker
Institute for Biomedical Engineering and Informatics
Technische Universität Ilmenau
98693 Ilmenau
eva-maria.doelker@tu-ilmenau.de

**Comments to the Academic Editor**

| |
|---|
| 1. Please ensure that your manuscript meets PLOS ONE's style requirements, including those for file naming. The PLOS ONE style templates can be found at https://journals.plos.org/plosone/s/file?id=wjVg/PLOSOne_formatting_sample_main_body.pdf and https://journals.plos.org/plosone/s/file?id=ba62/PLOSOne_formatting_sample_title_authors_affiliations.pdf. |
| Answer: We checked the style requirements and updated the manuscript accordingly. |
| 2. Please note that PLOS ONE has specific guidelines on code sharing for submissions in which author-generated code underpins the findings in the manuscript. In these cases, we expect all author-generated code to be made available without restrictions upon publication of the work. Please review our guidelines at https://journals.plos.org/plosone/s/materials-and-software-sharing#loc-sharing-code and ensure that your code is shared in a way that follows best practice and facilitates reproducibility and reuse. |
| Answer: Thanks for pointing out. We use only standard Matlab functions for standard statistics and standard visualization purposes which are explained in the manuscript. |
| 3. Thank you for stating the following financial disclosure: [copy in funding statement]. Please state what role the funders took in the study. If the funders had no role, please state: ""The funders had no role in study design, data collection and analysis, decision to publish, or preparation of the manuscript."" If this statement is not correct you must amend it as needed. Please include this amended Role of Funder statement in your cover letter; we will change the online submission form on your behalf. |
| Answer: We included the amended Role of Funder statement in our cover letter. |
| 4. We noted in your submission details that a portion of your manuscript may have been presented or published elsewhere. [Yes, In this paper, we report the second part of a |

| |
|---|
| set of experiments from a study on 81 volunteers where the first part is already published in 10.1038/s41598-022-10708-9.] Please clarify whether this publication was peer-reviewed and formally published. If this work was previously peer-reviewed and published, in the cover letter please provide the reason that this work does not constitute dual publication and should be included in the current manuscript. |
| Answer: We added an explanation in the cover letter. |
| 5. Please provide a complete Data Availability Statement in the submission form, ensuring you include all necessary access information or a reason for why you are unable to make your data freely accessible. If your research concerns only data provided within your submission, please write "All data are in the manuscript and/or supporting information files" as your Data Availability Statement. |
| Answer: We created a Zenodo repository (10.5281/zenodo.12800471), which will be set public upon acceptance of the paper. |

**Comments to the Reviewers**
**Reviewer #1: Overall, this is a clear, manuscripte, and well-written manuscript.**

This is an interesting study and the first impression about your article is stated below:

1. The manuscript is properly organized.
2. The abstract is well written and gives a reasonable summary for the article.
3. The introduction adequately covers the research issue
4. The researcher was successful in presenting the research methodology in detail, starting with the stages of preparing the subjects, the method of arranging the electrodes, and the method of applying the electrical signals in this study.
5. Before accepting the manuscript, in my opinion, there are few suggestions need to be considered with some notes require your explanation:

a. There is a need to include more previous studies on the topic

| |
|---|
| Answer: We thank the reviewer for pointing this out and have included more previous studies on the topic in the introduction section in form of the following paragraph (Introduction, p. 4):<br><br>“Previous research also investigated the suitability of varying current stimulation amplitudes, pulse intervals, and stimulation locations within the field of neurofeedback. Parameters used for encoding information are e.g. pulse frequency [11,12], number of pulses [13], and pulse width [11] whereas a fixed amplitude was often used. The spatial discriminability depends on the temporal stimulation parameters [14] and spatial identification was improved if only one electrode pair was stimulated instead of two simultaneously [13].<br>Building on these findings, our current research explored the effectiveness of varying current stimulation amplitudes, pulse intervals, and stimulation locations specifically for providing warning information. These parameters need to be investigated separately since the application in electrical warning systems has different requirements compared to neurofeedback applications.” |

b. I suggest including a diagram that briefly explains the research methodology to facilitate the understanding for the reader.

Answer: We added Fig 1 to explain the experimental setup and arrangement of the electrodes.

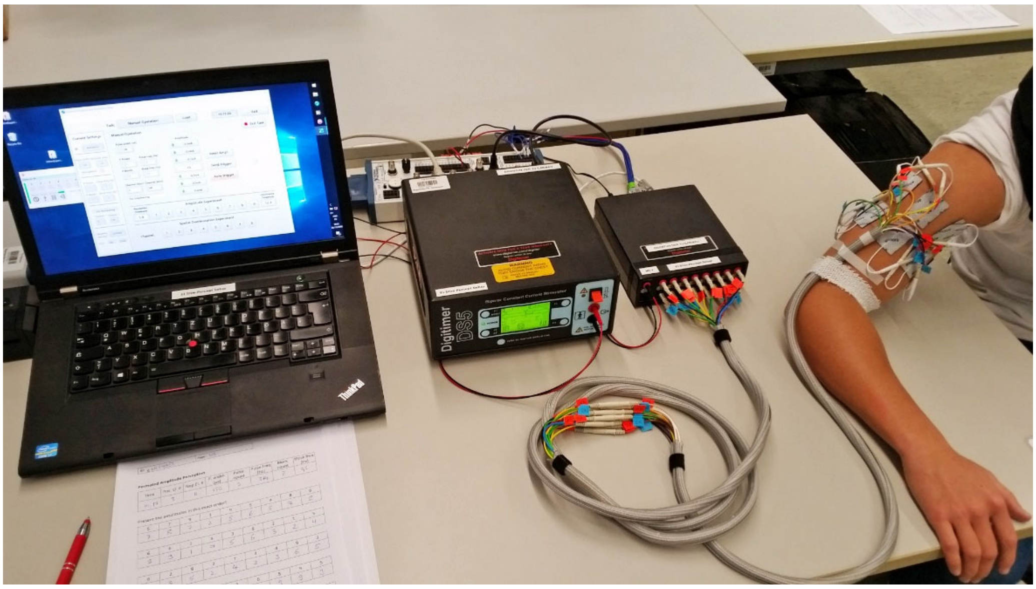


Fig 1. Photograph of the experimental setup. Left to right: Computer running LabVIEW, constant current stimulator DS5, multiplexer D188, and electrodes at the upper arm of one volunteer.

c. I suggest including figure showing the arrangement of the electrodes.

Answer: We added Fig 1 to explain the experimental setup and arrangement of the electrodes.

d. You mention the optimal impedance in the section on preparation. Is it necessary for the impedance to be higher or lower, and if so, why? Could you please explain how you arrived at the optimal impedance?

Answer: We are aiming for a low transition impedance in order to have a good electrical connection between the electrode and the skin. For that purpose, the skin of the participant was cleaned and moistened with a wet towel. To make this clearer, we changed the word optimal to the word low (Preparation, p. 7):

“To ensure a low impedance between the skin and electrodes, the participant's right upper arm was cleaned and moistened with a wet towel.”

**Reviewer #2:**

1. The introduction must highlight the main electrical stimulation basics and occupational safety.

Answer: Thanks for the suggestion, we added a paragraph in the introduction section addressing electrical stimulation basics and occupational safety requirements (Introduction, pp. 2-3):

"The electrocutaneous stimulation for the electrical warning system involves delivering controlled electrical impulses to the skin, creating tactile sensations typically without causing pain or discomfort.
For our electrical warning system, we envisioned several functional requirements. Firstly, as part of the personal safety equipment, the electrical warning signal must be perceivable, distinguishable from other stimuli, and unique. Secondly, the warning system should be capable of transferring a small number of signal types. Thirdly, the electrodes of the electrical warning system should be placed on a part of the body where the wearing comfort is high, the electrodes are easy to attach, the amount of sweat is relatively low, the restriction of motion is minimal, the potential for conflict with working and personal safety equipment is low, and the risk of muscle contraction and unexpected involuntary responses is relatively low.
In addition to these functional requirements, the electrical warning system could adhere to the ISO EN 60601-1 norm [4], which defines safety and ergonomic requirements for medical electrical devices and systems, as there is currently no specific norm for electrical warning systems. Lastly, usability is a major requirement for our electrical warning system. It should be well-tolerated by the user, wearable for up to 8 hours, lightweight, integrated into work clothes, and equipped with stable electronics and a reliable energy supply."

2. The contribution of the proposed work should be presented.

Answer: We thank the reviewer for pointing this out and have re-written the conclusion section to clearer present our contributions (Conclusion, pp. 15-16):

"Our study contributes stimulus interval values for the transition of the temporal perception between Pulsating, Vibrating, and Continuous. While these perceptions can be produced as needed and in this order across the pulse interval range, we found a high interindividual variability of the absolute pulse interval values. Therefore, the absolute values should be calibrated to the individual using the distributions reported in this study as a default starting point.
We established a linear relationship between presented and perceived amplitudes of the electric warning signals. This relationship might be used in structured warning signals and can serve as one means of coding information into this warning signal.
We established the discriminability of electric warning stimulation locations around the arm. This opens the possibility of encoding information about the type of warning into the signal in a workplace safety device. Given the high discriminability accuracy when taking the neighboring electrode pairs into account, four principal locations might be used for this information encoding.
In summary, this study underlined the realizability of an electric warning system for workplace safety and explored design dimensions of electrocutaneous stimulation signals at the upper arm with a biphasic current stimulation pulse during rest. For the design of an electrical warning signal, varying pulse intervals and stimulation locations (i.e. electrode pairs around the upper right arm) are directly applicable, while the stimulation amplitude may be incorporated in a structured stimulation pattern. Our research lays the groundwork for developing suitable signals for wearable electric warning devices."

3. The applicable guidelines and regulations the authors refer to in the Methodology Section need some focus, and the references related to these regulations must be inserted.

Answer: Thanks, we added the corresponding norm ISO EN 60601-1 and a paragraph within the introduction (Introduction, p.3):

"For our electrical warning system, we envisioned several functional requirements. Firstly, as part of the personal safety equipment, the electrical warning signal must be perceivable, distinguishable from other stimuli, and unique. Secondly, the warning system should be

capable of transferring a small number of signal types. Thirdly, the electrodes of the electrical warning system should be placed on a part of the body where the wearing comfort is high, the electrodes are easy to attach, the amount of sweat is relatively low, the restriction of motion is minimal, the potential for conflict with working and personal safety equipment is low, and the risk of muscle contraction and unexpected involuntary responses is relatively low.
In addition to these functional requirements, the electrical warning system could adhere to the ISO EN 60601-1 norm [4], which defines safety and ergonomic requirements for medical electrical devices and systems, as there is currently no specific norm for electrical warning systems. Lastly, usability is a major requirement for our electrical warning system. It should be well-tolerated by the user, wearable for up to 8 hours, lightweight, integrated into work clothes, and equipped with stable electronics and a reliable energy supply."

4. In the experimental work section, a block diagram and paragraph about the work should be added even if the author refers to previous relevant research

Answer: We added Fig 1 to explain the experimental setup and arrangement of the electrodes. We also added a paragraph to further explain the methods.

5. As the presented work is essential in occupational safety, what are the main challenges for the proposed work?

Answer: We added the functional challenges as well as other requirements as a paragraph into the introduction (Introduction, p.3):

"For our electrical warning system, we envisioned several functional requirements. Firstly, as part of the personal safety equipment, the electrical warning signal must be perceivable, distinguishable from other stimuli, and unique. Secondly, the warning system should be capable of transferring a small number of signal types. Thirdly, the electrodes of the electrical warning system should be placed on a part of the body where the wearing comfort is high, the electrodes are easy to attach, the amount of sweat is relatively low, the restriction of motion is minimal, the potential for conflict with working and personal safety equipment is low, and the risk of muscle contraction and unexpected involuntary responses is relatively low.
In addition to these functional requirements, the electrical warning system could adhere to the ISO EN 60601-1 norm [4], which defines safety and ergonomic requirements for medical electrical devices and systems, as there is currently no specific norm for electrical warning systems. Lastly, usability is a major requirement for our electrical warning system. It should be well-tolerated by the user, wearable for up to 8 hours, lightweight, integrated into work clothes, and equipped with stable electronics and a reliable energy supply."

6. The conclusion needs an improvement.

Answer: We thank the reviewer for pointing this out and have re-written the conclusion section to clearer present our contributions (Conclusion, pp. 15-16):

"Our study contributes stimulus interval values for the transition of the temporal perception between Pulsating, Vibrating, and Continuous. While these perceptions can be produced as needed and in this order across the pulse interval range, we found a high interindividual variability of the absolute pulse interval values. Therefore, the absolute values should be calibrated to the individual using the distributions reported in this study as a default starting point.
We established a linear relationship between presented and perceived amplitudes of the electric warning signals. This relationship might be used in structured warning signals and can serve as one means of coding information into this warning signal.

We established the discriminability of electric warning stimulation locations around the arm. This opens the possibility of encoding information about the type of warning into the signal in a workplace safety device. Given the high discriminability accuracy when taking the neighboring electrode pairs into account, four principal locations might be used for this information encoding.
In summary, this study underlined the realizability of an electric warning system for workplace safety and explored design dimensions of electrocutaneous stimulation signals at the upper arm with a biphasic current stimulation pulse during rest. For the design of an electrical warning signal, varying pulse intervals and stimulation locations (i.e. electrode pairs around the upper right arm) are directly applicable, while the stimulation amplitude may be incorporated in a structured stimulation pattern. Our research lays the groundwork for developing suitable signals for wearable electric warning devices."

**Reviewer #3:**

The manuscript titled "Design Dimensions of Electrocutaneous Warning Stimulation Patterns" addresses an important topic and presents valuable insights into the design dimensions of electrocutaneous warning stimulation patterns. With some revisions to enhance clarity, provide additional context, and strengthen the discussion, this study has the potential to make a significant contribution to the field.

1. The title effectively conveys the focus of the study on electrocutaneous warning stimulation patterns. However, it could be improved by specifying the specific context or application of these patterns to enhance clarity for readers.

Answer: We thank the reviewer for the suggestion and have changed the title accordingly: "Design dimensions of electrocutaneous warning stimulation patterns in workplace safety devices"

2. The abstract provides a concise summary of the study objectives, methods, and key findings. It effectively highlights the importance of the research but could benefit from more specific details on the experimental design and implications of the results.

Answer: We thank the reviewer for the suggestion and have elaborated (changes in red) the abstract accordingly (Abstract, p. 1):

"Warning of workers in dangerous situations is crucial. With the aim of deriving practical parameters for an electrocutaneous warning stimulation, we explore the design dimensions of pulse intervals, amplitudes, and locations of electrocutaneous stimulation in a study on healthy volunteers. Using biphasic rectangular current pulses on the upper right arm of 81 healthy participants, they evaluated temporal perception with varying intervals, ranging from 200 ms down to 0.5 ms, categorizing it as 'Individual pulses', 'Pulsating', 'Vibrating', or 'Continuous'. Next, we tested nine amplitude levels. Participants rated the perceived amplitude on a scale from 1 to 9 after a training phase. Finally, we presented five consecutive stimulation pulses in a pseudo-random order at eight electrode pair positions, asking participants to report the stimulated electrode pair. Participants perceived electrocutaneous pulses as 'Individual pulses' for median intervals above 74 ms, as 'Pulsating' between 44 ms and 74 ms, as 'Vibrating' between 12 ms and 44 ms, and as 'Continuous' below 12 ms. Pulse intervals below about 1 ms were perceived as weak and at about 5 ms as inconvenient, rendering these intervals less suitable for the design of a warning pattern. The median reported amplitudes [25%-75%-percentile] for presented amplitudes 1 to 9 are: 1[1-1], 2[2-3], 3[2-4], 3[3-4], 4[3-5], 5[4-6], 6[4-7], 7[5-8] and 7.5 [6-8] indicating a linear relationship between presented and perceived amplitude. These results

suggest that the stimulation amplitude may be incorporated into a structured stimulation pattern. The majority of the electrode pair locations were reported correctly (64.3% - 86.6%) or within the two neighboring electrode pairs (98% - 99.7%). We conclude that the determined pulse intervals combined with the differentiability of locations offer the basis for designing a warning signal. Our research lays the groundwork for developing suitable signals for wearable electric warning devices."

3. The introduction provides a clear rationale for the study by discussing the importance of optimizing electrocutaneous warning stimulation parameters for occupational safety. However, it would be beneficial to include more background information on existing research in this area to better contextualize the current study.

Answer: In order to contextualize the study, we added a paragraph in the introduction section including functional and other requirements for the warning system and more extensively reference existing research (Introduction, pp. 2-4):

"The electrocutaneous stimulation for the electrical warning system involves delivering controlled electrical impulses to the skin, creating tactile sensations typically without causing pain or discomfort.
For our electrical warning system, we envisioned several functional requirements. Firstly, as part of the personal safety equipment, the electrical warning signal must be perceivable, distinguishable from other stimuli, and unique. Secondly, the warning system should be capable of transferring a small number of signal types. Thirdly, the electrodes of the electrical warning system should be placed on a part of the body where the wearing comfort is high, the electrodes are easy to attach, the amount of sweat is relatively low, the restriction of motion is minimal, the potential for conflict with working and personal safety equipment is low, and the risk of muscle contraction and unexpected involuntary responses is relatively low.
In addition to these functional requirements, the electrical warning system could adhere to the ISO EN 60601-1 norm [4], which defines safety and ergonomic requirements for medical electrical devices and systems, as there is currently no specific norm for electrical warning systems. Lastly, usability is a major requirement for our electrical warning system. It should be well-tolerated by the user, wearable for up to 8 hours, lightweight, integrated into work clothes, and equipped with stable electronics and a reliable energy supply."

and

"Previous research also investigated the suitability of varying current stimulation amplitudes, pulse intervals, and stimulation locations within the field of neurofeedback. Parameters used for encoding information are e.g. pulse frequency [11,12], number of pulses [13], and pulse width [11] whereas a fixed amplitude was often used. The spatial discriminability depends on the temporal stimulation parameters [14] and spatial identification was improved if only one electrode pair was stimulated instead of two simultaneously [13].
Building on these findings, our current research explored the effectiveness of varying current stimulation amplitudes, pulse intervals, and stimulation locations specifically for providing warning information. These parameters need to be investigated separately since the application in electrical warning systems has different requirements compared to neurofeedback applications."

4. The methodology section is detailed and well-structured, providing a clear overview of the experimental design, participant characteristics, and data analysis procedures. However, additional information on the selection criteria for participants and the rationale behind the chosen stimulation parameters would enhance the methodological clarity.

Answer: We added a paragraph about the selection criteria of the participants and give the rationale behind the chosen stimulation parameters.

Line 87:
“Further exclusion criteria were heart disease or pacemaker, severe high blood pressure or hyperthyroidism, neurological or psychiatric pre-existing conditions, diabetes, numbness in the upper arm, implants in the right arm, significant skin diseases on the right upper arm, current or previous abuse of addictive substances, pregnancy or breastfeeding, and regular use of medication except contraception.”

Line 160:
“Thereafter, the pulse interval range was sampled to capture the range of the temporal perceptions and the transition pulse intervals.”

Line 168:
“To discretize the amplitude range with sufficient detail to quantify the participants’ accuracy while keeping the task feasible for a first-time user, the individual perception Ap and intolerance Ai thresholds have been used to generate 9 equally spaced values between 1.1·Ap and 0.9·Ai, where each amplitude was assigned to the values 1 to 9.”

Line 177:
“The amplitude sequence was chosen to include each amplitude six times and to provide similar quantities (3-4 instances) of the different amplitude step-sizes between successive stimuli, except for the most extreme step-sizes of +/-7 (2 instances each) and +/-8 (1 instance each), within a feasible sequence length. This supported the evaluation of the influence of the preceding stimulus on the perception of the current stimulus.”

Line 194:
“The sequence was chosen to include each position five times and each relative difference of successive positions in approximately even quantity (4-6 instances each), within a feasible sequence length.”

5. The results section presents the findings of the study in a logical sequence with appropriate statistical analysis. It effectively communicates the outcomes of varying pulse intervals, amplitudes, and electrode pair positions on temporal perception. Including visual aids such as tables or figures to supplement the text would further enhance the presentation of results.

Answer: We thank the reviewer for the suggestion. We have one Figure and/or Table for each result.

6. The discussion interprets the results in the context of the study objectives and compares them to existing literature. It effectively addresses the implications of the findings for occupational safety applications. However, expanding on the limitations of the study and suggesting potential avenues for future research would strengthen the discussion section.

Answer: We thank the reviewer for the suggestion and have expanded the discussion of the study limitations and outlined avenues for future research. The new paragraphs read:

“There are some limitations to our study. The investigated electrode configurations were limited to vertical pairs and a fixed electrode size, which was based on practical considerations. Other configurations like transversal or diagonal electrode pairs or the influence of the neighboring margin lengths of the electrodes should be investigated in future

studies. The experimental setup used in this study was designed primarily for baseline research in the laboratory. However, its stationary nature restricts its use in field studies and real-world warning scenarios. Future work will focus on miniaturizing the setup to make it portable, with the long-term goal of creating a wearable version for users. Further, the experiments have been conducted at the lateral electrode pair no. 3 with a pulse width of 150 µs (except for the spatial discrimination task). Although future studies need to investigate the effects of pulse width for all electrode positions, we believe that a pulse width of 150 µs can be chosen as a basic working parameter for a future warning system [9]. TENS electrodes were selected for this study due to their low and consistent impedance characteristics. However, they are not ideal for long-term use, as the gel can dry out over time, reducing their adhesive properties. In future work, textile electrodes will be further explored as a more suitable solution for wearable applications. In this study, a straightforward method of consecutively increasing the stimulation amplitude was used to determine perception, attention, and intolerance thresholds. This approach was chosen over more precise alternatives, such as the best PEST method [23], to maintain a manageable experimental duration and minimize participant fatigue or loss of concentration. The experimental paradigm was presented to all participants in the same order to ensure consistency across individuals. However, the absence of randomization may have introduced sequence effects. Gender differences reported here should be considered with caution since we had unequal sample sizes (29 females and 52 males). Young participants dominated the age distribution of the study group. Only 6 participants were older than 40 years. Consequently, future studies need to address the influence of age on the thresholds. Future studies will try to provide a more uniform distribution of participants between 18 and 65 years of age as other studies of electrocutaneous stimulation showed age-related effects on the perception threshold [18,24]. The majority of participants in this study were university students. Future research will focus on conducting experiments with employees, who represent potential users of the electrical warning system. Another limitation of this study is the lack of blinding, as the same individuals were involved in both conducting the experiments and evaluating the results, at least in part. In future work, these tasks will be separated to introduce a degree of blinding and reduce potential bias.“

7. The conclusion succinctly summarizes the key findings and their significance for optimizing electrocutaneous warning stimulation patterns. It effectively reinforces the study's contributions to the field of occupational safety.

Thank you for your comment.

8. It is essential to ensure that all ethical considerations, such as approval from relevant ethics committees and informed consent from participants, are clearly documented in the manuscript to uphold research integrity and compliance with ethical standards.

Thank you for your comment.
