# Supplementary material for: Design dimensions of electrocutaneous warning stimulation patterns in workplace safety devices
Source: PLoS One. 2024 Dec 16;19(12):e0314554. doi: 10.1371/journal.pone.0314554 (PMC11649102; doi:10.1371/journal.pone.0314554)
Supplement: Attachment — Submitted filename: Rebuttal letter_Re-Revision.pdf [file pone.0314554.s003.pdf]

Rebuttal letter

Dear Dr. Hadeel K. Aljobouri and Reviewers,

On behalf of my co-authors and myself, I extend our sincere thanks for your time and effort in reviewing our manuscript titled “Design dimensions of electrocutaneous warning stimulation patterns in workplace safety devices”. Your detailed comments and constructive feedback have been very helpful in improving our work. We appreciate your thorough evaluations and professional guidance.

Please find our responses to your comments below.

Best regards,

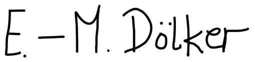

Dr. Eva-Maria Dölker
Institute for Biomedical Engineering and Informatics
Technische Universität Ilmenau
98693 Ilmenau
eva-maria.doelker@tu-ilmenau.de

Comments to the Academic Editor

1. Please review your reference list to ensure that it is complete and correct. If you have cited papers that have been retracted, please include the rationale for doing so in the manuscript text, or remove these references and replace them with relevant current references. Any changes to the reference list should be mentioned in the rebuttal letter that accompanies your revised manuscript. If you need to cite a retracted article, indicate the article’s retracted status in the References list and also include a citation and full reference for the retraction notice.
Answer: We checked the reference list accordingly in order to address the abovementioned queries.
2. Keywords must be included in the manuscript.
Answer: We included the keywords in the manuscript.
3. The number of references is minimal, they must be 30 minimum. We prefer to add more regarding the simulation, I recommend the authors read this paper as an example: “A Virtual EMG Signal Control and Analysis for Optimal Hardware Design,” International Journal of Online and Biomedical Engineering (iJOE), vol. 18, no. 02, pp. 154–166, Feb. 2022, doi: 10.3991/IJOE.V18I02.27047. However, there is no need to cite the paper.
Answer:<br>We added the following paragraph in the Introduction section:<br><br>Noninvasive electrical stimulation is broadly applied in areas such as muscle stimulation [5–8] and transcutaneous electrical nerve stimulation (TENS) [9–11], as well as in specialized uses like electrical acupoint stimulation to reduce postoperative nausea and vomiting [12]. Recent studies on electrical muscle stimulation have explored the impact of muscle length on torque generation [6], its effects on visual sensory reweighting dynamics [5], and muscle metabolism assessment [7]. TENS has been studied for pain management [9,10] and reducing postoperative wound infections [11].<br><br>And the following sentence in the discussion:<br>The occurrence and intensity of muscle twitches during electrical stimulation are influenced by individual anatomical factors, such as muscle length, with longer muscles producing

greater torque during stimulation [6]. These individual variations should be taken into account in the continued development of the electrical warning system.

This includes the following new references:

5. Shindo M, Isezaki T, Koike Y, Aoki R. Induced effects of electrical muscle stimulation and visual stimulation on visual sensory reweighting dynamics during standing on a balance board. PLOS ONE. 2023; 18:e0285831. doi: 10.1371/journal.pone.0285831.
6. Cavalcante JGT, Ribeiro, Victor Hugo de Souza, Marqueti RdC, Paz IdA, Bastos JAI, Vaz MA, et al. Effect of muscle length on maximum evoked torque, discomfort, contraction fatigue, and strength adaptations during electrical stimulation in adult populations: A systematic review. PLOS ONE. 2024; 19:e0304205. Epub 2024/06/10. doi: 10.1371/journal.pone.0304205 PMID: 38857245.
7. Tanaka R, Shigemori Y, Moriguchi T. Non-Invasive Muscle Metabolism Assessment with Near-Infrared Spectroscopy and Electrical Muscle Stimulation. BioMed. 2024; 4:419–29. doi: 10.3390/biomed4040033.
8. Ibitoye MO, Hamzaid NA, Ahmed YK. Effectiveness of FES-supported leg exercise for promotion of paralysed lower limb muscle and bone health-a systematic review. Biomed Tech (Berl). 2023; 68:329–50. Epub 2023/03/01. doi: 10.1515/bmt-2021-0195 PMID: 36852605.
9. R. M. Amaral A, C. Araújo R, A. Amorim D, A. Valença A, R. Pitangui AC. High and low frequency TENS in young people with primary dysmenorrhea: Clinical trial protocol. mtprehabjournal. 2024; 22. doi: 10.17784/mtprehabjournal.2024.22.1361.
10. Jha MS, Ahluwalia R, Chugh T, Gupta AR. Effectiveness of TENS and Acetaminophen for Pain Control During Orthodontic Treatment: A Comparative Study. Journal of Pharmacy and Bioallied Sciences. 2024; 16:S2916-S2918. doi: 10.4103/jpbs.jpbs_578_24.
11. Qin Y, Liu CL, Liu L. The role of TENS therapy in reducing the incidence of post-Caesarean section wound infection. International Wound Journal. 2024; 21. doi: 10.1111/iwj.70001.
12. Lu L, Xie C, Li X, Zhou Y, Yin Z, Wei P, et al. Efficacy and safety of electrical acupoint stimulation for postoperative nausea and vomiting: A systematic review and meta-analysis. PLOS ONE. 2023; 18:e0285943. doi: 10.1371/journal.pone.0285943.
